# Recovery of knee extension and incidence of extension deficits following anterior cruciate ligament injury and treatment: a systematic review protocol

**DOI:** 10.1186/s13018-019-1127-8

**Published:** 2019-03-28

**Authors:** Nalan Ektas, Corey Scholes, Selin Kulaga, Garry Kirwan, Binglong Lee, Christopher Bell

**Affiliations:** 1 EBM Analytics, Sydney, NSW Australia; 2 Physiotherapy Department, Queen Elizabeth II Jubilee Hospital, Brisbane, QLD Australia; 3 Orthopaedics Department, Queen Elizabeth II Jubilee Hospital, Brisbane, QLD Australia; 4 Menzies Health Institute, School of Allied Health Sciences, Griffith University, Gold Coast, Australia

## Abstract

**Introduction:**

Knee extension deficit or loss of extension (LOE) is a potential complication following ACL reconstruction (ACLR); however, the change in postoperative knee extension during rehabilitation is not well defined. The aim of this review is to establish the trajectory of knee extension recovery and incidence of knee extension deficit during rehabilitation after ACL rupture.

**Methods and analysis:**

A systematic search will be conducted in MEDLINE, Embase, Cochrane Library, Scopus, SPORTDiscus, and relevant trials databases of English language papers in publication as of May 2018, with no restrictions on publication year applied. References will be screened and assessed for eligibility by two independent reviewers as per the PRISMA guidelines. Cohort, cross-sectional or case-controlled studies will be included for the analysis. Data extraction will be conducted using a predefined template and quality of evidence assessed. Statistical summaries and meta-analyses will be performed as necessary.

**Ethics and dissemination:**

This review will provide clearer definitions for the measurement and interpretation of postoperative knee extension and establish its natural history after ACL reconstruction. Evidence of the incidence and factors associated with loss of extension will be identified. The findings of this systematic review will be disseminated in peer-reviewed journals and presented at national/international conferences.

**Trial registration:**

The protocol was registered on the PROSPERO international prospective register of systematic reviews prior to commencement (registration number CRD42018092295).

## Article summary

- To the best of our knowledge, this is the first systematic review examining the incidence and recovery of knee extension deficits at early and late phases of recovery following ACL treatment.
- Stringent methods will be applied during the review process to ensure quality of studies included for analysis.
- A limitation of this systematic review is that studies in English will be included only.
- This is a protocol for a systematic review; the results of the review will be published separately.

## Introduction

Loss of the ability to fully straighten the knee is a potential complication of ACL rupture and treatment, known to occur following surgical reconstruction [1, 2]. While it can be resolved with additional intervention [3], it remains a common cause of failure and revision surgery [4], as patients tend to be less tolerant of extension loss than loss of flexion [5]. Nevertheless, loss of extension (LOE) has also been reported in patients electing to undergo nonoperative treatment (1% incidence) [6]. Lack of full extension following ACL rupture may contribute to functional deficits and an increased risk of osteoarthritis [7]. However, the point at which extension loss becomes detrimental is not clearly understood.

LOE in this context has a complex aetiology, ranging from soft-tissue fibrosis, capsular adhesions, or technical errors during surgery (e.g., tunnel misplacement) leading to graft impingement [8]. Other generalisable risk factors include involvement of other knee structures, preoperative range of motion, and poor rehabilitation compliance [5]. While the degree of knee extension at early follow-up (4 weeks) is strongly related to extension loss at 12 weeks follow-up [9], the reported incidence of LOE is variable. One study reported a reoperation rate due to LOE of 5% in the first year after reconstruction [10], while another reported an incidence of stiffness of 12% at 6 months follow-up, but did not specify extension or flexion [5]. Others have reported LOE at 4 weeks of 25.3% in 229 patients [11]. However, the variable incidence rates may be attributed to varying methods of defining, detecting and classifying extension loss between studies.

The ability to straighten the knee without muscle contraction (passive) does not necessarily mean a patient will use the entire range of motion during loading (active extension), such as locomotion. One study reported reduced extension in patients with an ACL-deficient knee [12] and after reconstruction [13] during treadmill walking compared to controls. If ACL deficient or reconstructed knees impact the ground during heel strike of gait, where loads rapidly peak well above bodyweight in < 0.5 s, it may alter the loading patterns through the tibiofemoral cartilage and instigate degenerative changes [14]. Despite the volume of evidence produced on ACL injury, treatment and surgical reconstruction, recent reviews of ACL outcomes, particularly from a surgical perspective, have summarised peak knee flexion during locomotion [15, 16] or have focused on patient-reported subjective outcomes, joint stability or onset of osteoarthritis [17]. The ability to achieve full extension, particularly under active load, potentially links many of these outcomes; however, the trajectory of its recovery after injury and treatment remains relatively unknown.

The incidence of patients presenting with measurable LOE remains largely unknown. The difficulty in establishing a benchmark for treatment is due to the large range of options spanning from nonoperative to arthroscopic repair with additional procedures such as arthroscopic cartilage restoration, meniscal or combined ligament or cartilage repair. There is a lack of consistent information in the literature to establish a reasonable benchmark for future efforts with respect to reducing the incidence of extension loss and to encourage full use of active extension in patients diagnosed with ACL rupture. .To address this gap in the current knowledge, the primary objective of this review is to describe, in patients diagnosed with ACL rupture electing to undergo formal treatment (operative or nonoperative), compared to the non-operated limb, or to uninjured patients, the recovery of minimum knee extension angle measured under passive (no muscle contraction) and active (during locomotion) conditions. The secondary objectives are to explore the definition and incidence of LOE at early (3 months) and late (6 months—2 years) stages of recovery.

## Methods and analysis

The protocol was registered on the PROSPERO International Prospective Register of Systematic Reviews, registration number CRD42018092295. The systematic review follows the Preferred Reporting Items for Systematic Review and Meta-analysis (PRISMA-P) guidelines [18].

### Eligibility criteria

Relevant characteristics for included studies were determined using the PICOS (Population, Intervention, Comparison, Outcomes, Study Design) framework for formulating the research question and defining eligibility criteria for the literature search [19].

#### Population (inclusion/exclusion criteria)

All adults diagnosed with ACL rupture will be considered for review, without exclusions relative to patient sex, activity level or age.

The exclusion criteria for study selection will be:

- Participants diagnosed with multiple-ligament rupture or patellar dislocation secondary to ACL rupture
- Paediatric cases (patient aged < 18 years at time of surgery)

#### Intervention method

- Arthroscopic reconstruction of the ACL using autograft or allograft with any preparation and independent drilling of the femur and tibia and anatomical graft placement (footprint centre to footprint centre)
- Conservative management using
  ○ Surgical repair of the ligament or
  ○ Injectable therapies (e.g. platelet-rich-plasma)
  ○ Rehabilitation or exercise therapy

The exclusion criteria for study selection will be:

- Patients undergoing revision ACL reconstruction
- ACL reconstruction or repair procedures associated with joint preserving surgery for unicompartmental degenerative disease treated with
  ○ Tibial or femoral osteotomy
  ○ Meniscus transplantation
  ○ Meniscal prosthesis implantation
  ○ Joint arthroplasty

#### Comparators

- Contralateral limb of patients with ACL rupture, or
- Patients unaffected by ACL rupture (healthy controls)

#### Outcomes included for the review will be the following

- Passive knee extension (°) (no muscle contraction). Studies that have conducted measurements while patients are supine without muscle contraction to achieve minimum knee flexion.
- Active knee extension (°) (involving muscle contraction). Studies that have included measurements while patients conducted locomotion tasks (walking, running, stair climbing, landing on either one or both legs) as a minimum value during the movement or at the instant of ground impact.
- Incidence of knee extension deficit (%)—when the index knee is compared to the contralateral knee.
- Incidence of knee extension loss (%) in longitudinal, repeated measures studies that have compared post intervention knee extension to the pre-intervention value for the same patients.

#### Study designs included for review will be the following

- Observational studies (cohort, cross-sectional and case-controlled prospective or retrospective studies) or randomised controlled trials (RCTs) that compare the loss of extension or extension degeneration following ACLR with a minimum follow-up of 12 weeks. Systematic reviews will be used to source additional primary materials but will not be included in the analysis. The results of meta-analyses will be included as a study in the analysis if they meet the remaining inclusion criteria. English language papers in publication will be included, with no restrictions on publication year.

### Information sources

A systematic search will be conducted in PubMed for MEDLINE, Embase via Ovid SP, Cochrane Library, Scopus and SPORTDiscus via EBSCO and relevant clinical trials databases of English language papers in publication as of May 2018, with no restrictions on publication year applied (EBSCO, AMED, CINAHL, SPORTDiscus, EMBASE, Cochrane, LILACS, MEDLINE, PEDro, Scielo, SCOPUS & Web of Knowledge). Secondary searching of reference lists of key articles and grey literature will be undertaken in order to identify any additional studies potentially missed in electronic search. Active researchers in the field will be contacted to ensure relevant references have been captured.

### Search strategy

**Fig. 1:**
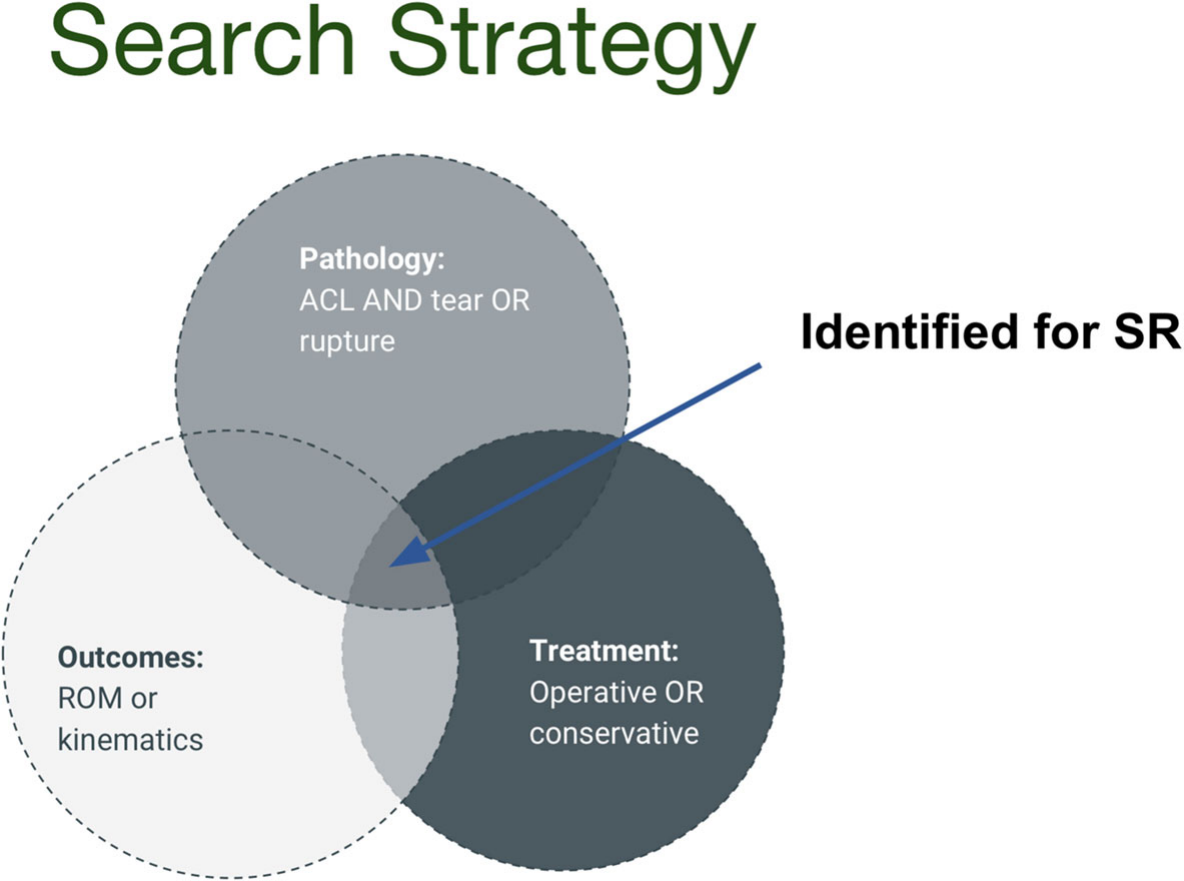
Search strategy for the systematic review

In order to permit the search to return other primary studies that were not included to the published reviews, medical subject headings (MeSH) terms and keywords such as systematic review, review and meta-analysis will be excluded. The main key domains are (1) pathology, (2) intervention and (3) outcomes of interest (Fig. 1). The main MeSH keywords are anterior cruciate ligament, ACL, knee, rehabilitation, physiotherapy, surgical, range of motion, extension and stiffness. Keywords within concept areas will be mutually inclusive (via ‘OR’ operator) and will be combined with the other key areas using an ‘AND’ operator as previously described [20].

The search will be comprised of the following components, which will be performed individually prior to filtering for duplicate records and preliminary analysis:

1. Biomechanics (ACL OR ‘anterior cruciate ligament’) AND (walk* OR jog* OR run* OR locomot* OR ambulat* OR stair* OR gait* OR stop* OR land* OR hop) AND (kinemat* OR biomech*) NOT (paediatric OR immature OR revision* OR revised)
2. Clinical (outcomes) (ACL OR ‘anterior cruciate ligament’) AND (extension OR ‘fixed flexion’ OR ‘loss of extension’ OR function OR ‘extension deficit’ OR ‘extension loss’ OR stiffness OR arthrofibrosis OR impingement OR ‘minimum flexion’ OR ‘range of motion’) NOT (paediatric OR immature OR revision* OR revised OR injury OR prevention)
3. Treatment (ACL OR ‘anterior cruciate ligament’) AND (rehab* OR therapy OR nonoperative OR management OR conservative OR surgical OR surgery OR repair OR reconstruction OR graft OR arthroscop*) NOT (paediatric OR immature OR revision* OR revised OR injury OR prevention)

Further information about the search used for MEDLINE is included in Appendix. The search strategy will be adjusted for application to other databases as appropriate. Search results will be supplemented by drawing relevant articles from

1. References lists from included studies, prioritising systematic reviews and meta-analyses
2. Clinical trial reports from Cochrane central register of controlled trials, Australia and New Zealand clinical trials register, Clinicaltrials.gov, World Health Organisation international clinical trials registry

### Study records

The study search and selection process will be based on the four-phase PRISMA flow process [21] for identification, screening, assessment of eligibility and inclusion of studies for the systematic review. A web-based bibliographic software package (Paperpile LLC, Vienna, Austria) will be used for data management. Citations and abstracts identified during the study search will be imported to the bibliographic software and duplicates removed. The study selection process will be performed independently by two reviewers. Title and abstract screening will be performed and full text files will be retrieved and uploaded to the reference software. Eligible studies will be identified for inclusion in the review. Data extracted and synthesised by the two independent reviewers will be author names, publication years, design of the included primary studies, inclusion criteria for primary studies, group intervention and comparison of the primary studies, tools used for outcomes assessment, the outcomes of interest and references of the primary studies.

Customised forms will be used for assessment of eligibility during the selection process and extraction of data. Consensus for inclusion and data extraction will be established amongst co-authors prior to review commencement, with study eligibility and data extraction forms piloted by each reviewer prior to use. Where agreement for study inclusion or data extraction is unable to be reached by the two reviewers, a third reviewer from the study team will be consulted.

### Data items

Study, parameters, population characteristics, as well as clinical, biomechanical and surgical factors will be extracted from included studies. Data relating to factors that may influence the change in and occurrence of knee extension loss will also be extracted from included studies. Data items for extraction (Table 1) include details of study characteristics, patient and surgical factors, as well as outcomes of interest. Data will be extracted and synthesised by two independent reviewers including author names, publication years, study design, inclusion criteria, intervention and comparison of the primary studies, methods used for outcomes assessment, results for the variables of interest (i.e. ROM, functional scores) and references of the primary studies.

**Table 1:** Data extraction items

Study items	First author	Year of publication
Study characteristics	Study design	Number of participants
	Follow-up period	
Patient	Age	Gender
	BMI	Diagnosis
	Sport or activity level of participation	Previous surgery
	Concomitant injuries	Comorbidities
Intervention	Intervention/treatment	Timeline of intervention
	Surgical technique/fixation	Graft type
	Graft preparation	Bone tunnel placement method
	Concomitant injury management	Bone tunnel placement strategy/philosophy
Comparators	Comparator group	
Outcomes	Knee extension (°)	Measurement method
	Timing of data collection	Movement paradigm
	Knee extension loss definition	Knee extension loss (%)
	Statistical tests used	

### Risk of bias

Independent scoring of risk of bias for included studies will be performed by two reviewers, with consensus reached by discussion. The ROBINS-I (Risk of Bias In Non-randomised Studies-of Interventions) tool [22] will be used to assess risk of bias in the observational studies eligible for inclusion. Potential risks will be assessed over seven bias domains: baseline confounding, participant selection, classification of intervention, deviations from intended intervention, missing data, outcomes measurement and reporting [22]. Bias due to confounding will be determined if one or more prognostic variables predicts the baseline intervention. Risk of bias for study participation will assess the likelihood of associations between intervention and outcomes, when the initial follow-up time of participants or outcome events are missing or excluded from some or selected eligible participants. Assessment of misclassification of intervention status (either differential or non-differential) will be used to determine bias in classification of intervention. Bias due to deviations from intended interventions will examine the likelihood of systematic differences between the experimental and proposed intervention at baseline. Missing data risks will be determined in the event follow-up data is missing for individuals initially included and followed in the study. Bias introduced by errors in measurement of outcome data (either differential or non-differential) will also be assessed. Selective reporting of results will be used to determine risk of bias for reporting. An overall risk of bias judgement will be determined as either low, moderate, serious or critical risk of bias or no information, for each specified outcome. Where more than one outcome of an included study is to be assessed, the risk of bias across the seven domains will be repeated for each key outcome, and a risk of bias judgement will be reported for all outcomes.

### Data synthesis and meta-analysis

Where the same outcome has been reported across multiple studies, a quantitative synthesis will be conducted. Data from included studies will be loaded into Review Manager (v5.3) (Mazuquin et al. 2018) and heterogeneity index (*I*^2^) will be calculated [23]. Where required, angles reported from studies will be converted to flexion angles, with negative angles indicating hyperextension of the knee and fixed flexion denoted by positive angles at the minimum flexion position. A graphical assessment of publication bias will be performed using a funnel plot and Begg’s test conducted as a statistical assessment [24].

A meta-analysis is planned to answer the questions: What is the recovery of knee extension after ACL rupture and treatment? What factors are associated with knee extension angle or loss of extension? Summary and descriptive statistics will be reported in terms of means, standard deviations, medians and ranges, as appropriate [25].

A meta-regression will be performed on the outcomes using patient, intervention, measurement and study characteristics as predictors. Subgroup analyses will be performed where data is sufficient to assess the influence of patient or surgical factors on reported knee extension and incidence of knee extension deficits:

- Time from surgery to measurement of knee extension (recovery trajectory)
- Intervention (reconstructed versus conservative management)
  ○ Surgical technique variations in reconstructed patients (graft type, fixation, tunnel drilling, notchplasty)
- Control group (contralateral, non-injured / non-operated)
- Movement paradigm (walking, running, landing)
- Measurement method (clinical judgement, goniometry, inertial sensors, optical motion capture)

Where quantitative synthesis is not appropriate, the extracted data will be summarised in tables and narrative interpretation provided, with particular emphasis on operational definitions of extension loss and measurement methods of knee extension angles. Publication bias will be assessed using funnel plots with standard error of incidence of loss of extension, or knee extension angle during either passive or active tasks. Where required, mirroring of low-sample studies will be used to enable visualisation.

### Confidence in cumulative evidence

The revised and validated Methodological Index for Non-Randomised Studies (MINORS) Criteria [26] will be used to assess the strength of non-randomised studies included for the review. The MINORS tool applies a scoring system across 12 items to assess the methodological and scientific value of studies, with the first 8 items relating to non-comparative studies and all 12 items relevant for comparative studies. Each item will be scored from 0 to 2, with 0 indicating a lack of reporting of the item, 1 indicating inadequate reporting and 2 indicating adequate reporting of the item in the evaluated study with maximum scores for non-comparative and comparative studies of 16 and 24 respectively. The MINORS score for non-randomised studies will be categorised as per [3]; 0 < MINORS score < 6 to indicate a very low-quality evidence, 6 ≤ MINORS score < 10 to indicate low quality of evidence, 10 ≤ MINORS score < 14 to indicate fair quality of evidence and MINORS score > 15 to indicate good quality of evidence. Where randomised controlled trials are included, in the context of a primary comparison between alternative interventions (e.g. surgical vs non-surgical management of ACL rupture) with respect to the review outcomes, the GRADE system will be utilised to assess study quality [27].

### Documenting protocol amendments

Protocol amendments and updates will be documented via PROSPERO online register. The nature of the changes made will be recorded, dated and accessible along with the most recent version within the record audit trail under the systematic review protocol registration number CRD42018092295.

## Appendix

Search strategy MEDLINE (via Ovid)

1. ACL
2. Anterior cruciate ligament/
3. Rupture/
4. 1 or 2
5. 3 and 4
6. Reconstructive surgical procedures/
7. Anterior cruciate ligament reconstruction
8. Exercise therapy
9. Physical therapy modalities/
10. Biological therapy/
11. or/6-10
12. Knee/
13. Knee joint/
14. 12 or 13
15. Extension
16. Range of motion, articular/
17. Extension deficit
18. Gait
19. Biomechanic$
20. Kinematic$
21. or/15-20
22. 5 and 11 and 14 and 21

Ovid
